# Bilateral chorea due to hemodynamic ischemia associated with bilateral internal carotid artery stenosis: A case report

**DOI:** 10.1111/cns.14070

**Published:** 2022-12-27

**Authors:** Jiali Zhao, Fudi Chen, Ruirui Yang, Chunxia Li, Lin Lu

**Affiliations:** ^1^ Department of Neurology Shandong Provincial Hospital Affiliated to Shandong First Medical University Jinan Shandong China; ^2^ Department of Emergency Shandong Provincial Hospital Affiliated to Shandong First Medical University Jinan Shandong China

Dear Editor,

Chorea is defined as rapid, involuntary, non‐rhythmic, non‐stereotypical movements originating from dysfunctional neuronal networks interconnecting the basal ganglia and frontal cortical motor areas.^1^ Bilateral chorea is a very rare manifestation with cerebrovascular factors. To date, bilateral chorea related to stenosis of the bilateral internal carotid artery (ICA)has not been reported. We encountered a 74‐year‐old woman presented with volatile bilateral limbs involuntary movement associated with bilateral ICA stenosis. Her DWI images revealed no lesion on basal ganglia, but CTP showed hypoperfusion in bilateral cerebral hemispheres. Our case report supports t that hemodynamic ischemia may be the key factor in bilateral chorea, which is associated with bilateral internal carotid artery stenosis. Clinicians should not misdiagnose bilateral chorea as other neurological disorders such as Huntington's disease or small chorea.

## 1 CASE REPORT

A 74‐year‐old woman was presented to our hospital because of volatile bilateral limbs involuntary movement for 40 days. The initial chorea involved only right upper extremities and oral dyskinesia, which was volatile. The symptoms gradually worsened 30 days ago and developed continuous involuntary movements of all four extremities. she was a non‐smoker. There was no history of hypertension, diabetes mellitus, cardiac arrhythmia, or prior neuroleptic exposure. She had no significant family history.

On admission, the patient had clear consciousness. Neurological examination showed normal ocular movements and continuous oral dyskinesia. Her limbs had no weakness. Her four extremities pronated and supinated, and his fingers twisted. His gait was slightly disturbed because of clumsy involuntary movements. She was diagnosed as chorea by a neurologist.

**FIGURE 1:**
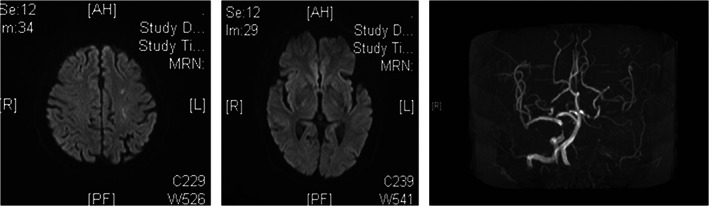
DWI images only showing a minimum high‐intensity lesions in the left frontal lobe and MRA revealed severe stenosis or occluded of the left ICA and stenosis of the right ICA.

**FIGURE 2:**
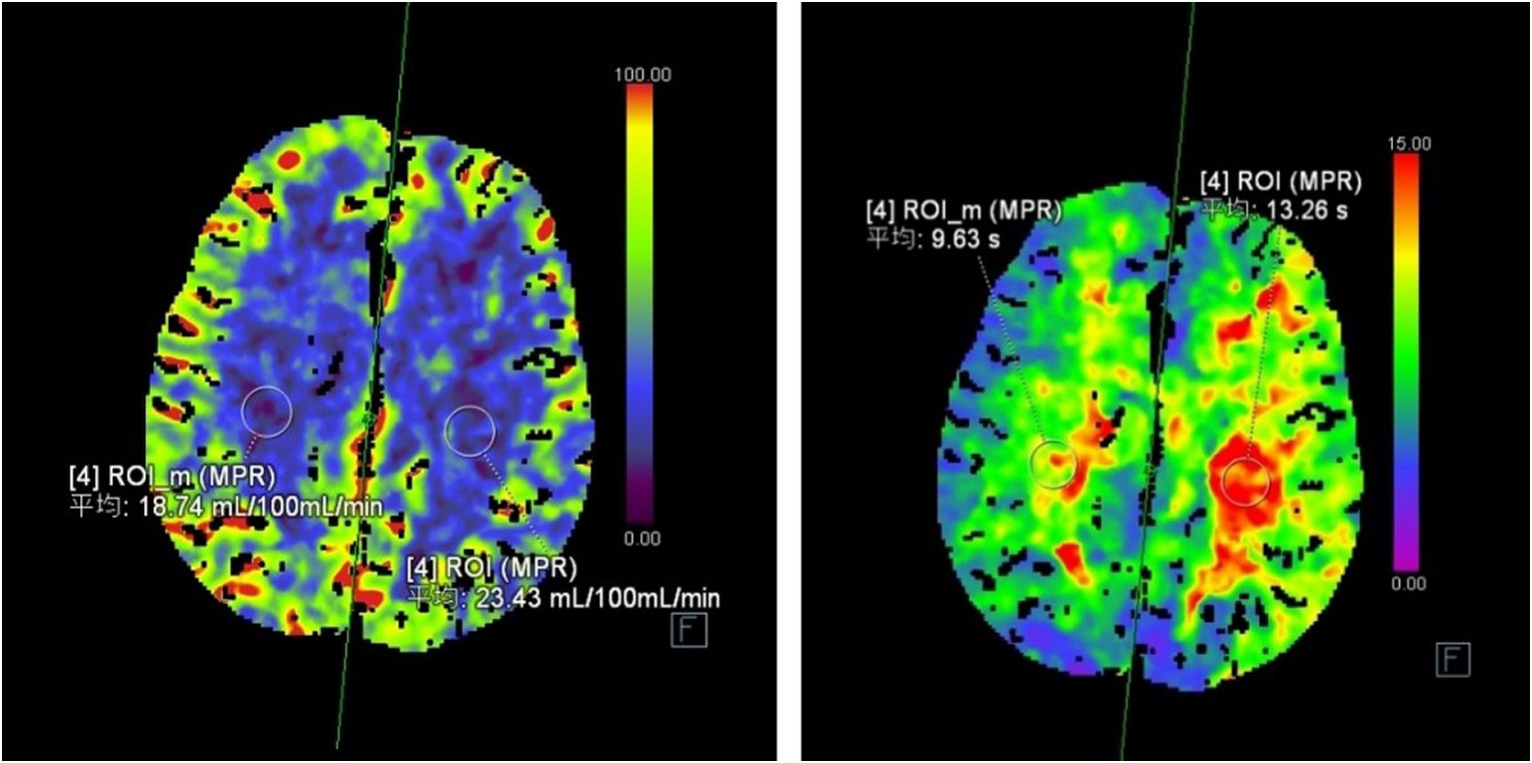
Brain CTP showed hypoperfusion

Results of laboratory tests including blood cell count, urinalysis, glucose, glycated hemoglobin, thyroid function, serum copper, and ceruloplasmin were unremarkable. Tumor markers were also unremarkable. There was no spiny red blood cell. Immunologic tests, including ASO, RF, ESR, antinuclear, immunoglobulin electrophoresis, antineuronal antibodies, and autoimmune encephalitis antibodies were negative. Cerebrospinal fluid test including cell count, protein level, glucose level, India ink, and exfoliative cytology were normal. DWI images only revealed a minimum high‐intensity lesions in the left frontal lobe and no lesion on basal ganglia. Meanwhile, magnetic resonance angiography (MRA) revealed severe stenosis or occluded of the left ICA (internal carotid artery) and stenosis of the right ICA (Figure 1). Brain CT perfusion imaging showed left frontal, temporal lobe, right semi‐oval area, and right basal ganglia cerebral blood flow (CBF) reduction (Figure 2). The symptoms did not improve with haloperidol and sulpride; however, bilateral chorea gradually subsided after administered clopidogrel, rosuvastatin, and butylphthalide. After the 6‐month follow‐up, the patient reported no residual symptoms.

## 2 DISCUSSION

Chorea is one of the major types of involuntary movement disorders originating from dysfunctional neuronal networks interconnecting the basal ganglia and frontal cortical motor areas.^1^ Cerebrovascular insults, neoplasms, infections, metabolic disorders, neurodegenerative disorders, and immunological diseases are known etiologies.^2^ However, chorea is a rare complication of acute vascular lesions.^1^ Yuki Ueta et al^3^ reported a case of persistent hemichorea as a preceding of cerebral infarction due to right middle cerebral artery stenosis. However, bilateral chorea related to severe stenosis of the bilateral ICA has not been reported.

We encountered a patient with bilateral ICA stenosis presenting with bilateral chorea for 40 days. Although this case lacks ischemic lesion on basal ganglia with only a minimum high‐intensity lesions in the left frontal lobe in DWI imaging, there is evidence of severe stenosis or occluded of the bilateral ICA. CTP showed hypoperfusion in bilateral cerebral hemispheres (CBF < 25 ml/100 ml/min),^4^ which supports the ICA stenosis diagnosis. However, CTP is by no means a specific methodology for the diagnosis, as decreased cerebral metabolic rate of oxygen has been reported in patients with Huntington's disease.^5^ In addition, our patient did not achieve good results with haloperidol and sulpride. However, the symptoms improved after clopidogrel, rosuvastatin, and butylphthalide. we speculate that the patient's bilateral chorea was caused by hypoperfusion in the striato‐thalamic‐cortex caused by stenosis of the bilateral ICA. The bilateral motor cortico‐striato‐pallido‐thalamo‐cortical loop and a functional disconnection might have been resulted in the chorea of all four extremities^6^ in our patient. In future studies, other imaging modalities, such as UCB‐J PET of synaptic SV2 protein density in the striatum should be considered.^7^

In conclusion, bilateral chorea maybe a syndrome of hemodynamic ischemia that should not be misdiagnosed as other neurological disorders such as Huntington's disease or small chorea. Clinicians should consider the existence of underlying bilateral carotid stenosis or occlusive disease with bilateral chorea.

## 3 AUTHOR CONTRIBUTIONS

Jiali Zhao collected and evaluated the data and drafted and revised the manuscript. Fudi Chen reviewed the literature. Jiali Zhao, Ruirui Yang, Chunxia Li, and Lin Lu examined and evaluated the patient. All authors read and approved the final manuscript.

## 4 CONFLICT OF INTEREST

The authors declare that they have no conflict of interest.

## 5 INFORMED CONSENT

Informed consent was obtained from all individual participants included in the study.

## Data Availability

All data generated or analyzed during this study are included in this published article.
